# Low-Cost High Performance Polyamide Thin Film Composite (Cellulose Triacetate/Graphene Oxide) Membranes for Forward Osmosis Desalination from Palm Fronds

**DOI:** 10.3390/membranes12010006

**Published:** 2021-12-22

**Authors:** Tarek S. Jamil, Rabab A. Nasr, Hussien A. Abbas, Tamer I. M. Ragab, Sinethemba Xabela, Richard Moutloali

**Affiliations:** 1 National Research Center El Behouth Street Dokki, Water Pollution Control Department, Dokki 12622, Cairo, Egypt; omaytarek73@yahoo.com; 2 National Research Centre, Inorganic Chemistry Department, El Behouth Street Dokki, Dokki 12622, Cairo, Egypt; hu_abbas2005@yahoo.com; 3 National Research Centre, Chemistry of Natural and Microbial Products Department, Dokki 12622, Cairo, Egypt; tamerragab2006@gmail.com; 4 Department of Chemical Sciences, University of Johannesburg, Doornforntein 2028, South Africa; sinethembaxabela@gmail.com; 5 Campus Florida, Institute for Nanotechnology and Water Sustainability, College of Science, Engineering and Technology, Florida Science, University of South Africa, Johannesburg 1709, South Africa; moutlrm@unisa.ac.za

**Keywords:** cellulose triacetate, palm fronds, forward osmosis, thin-film composite membranes, graphene oxide

## Abstract

Novel low-cost cellulose triacetate-based membranes extracted from palm fronds have been fabricated through the phase–inversion procedure. The cellulose tri-acetate (CTA) membrane was modified by incorporation of graphene oxide (GO) prepared from palm fronds according to the modified Hummer method as well as the preparation of polyamide thin film composite CTA membranes to improve forward osmosis performance for seawater desalination. The surface characteristics and morphology of the prepared CTA, GO, and the fabricated membranes were investigated. The modified TFC prepared membrane had superior mechanical characteristics as well as permeation of water. The performance of the prepared membranes was tested using synthetic 2 M Sodium chloride (NaCl) feed solution. The water flux (J_w_) of the thin-film composite (TFC) (CTA/0.3% GO) was 35 L/m^2^h, which is much higher than those of pure CTA and CTA/0.3% GO. Meanwhile, the salt reverse flux TFC (CTA/0.3% GO) was 1.1 g/m^2^h), which is much lower than those of pure CTA and CTA/0.3%. GO (Specific salt flux of TFC (CTA/0.3% GO) substrate membrane was 0.03 g/L indicating good water permeation and low reverse salt flux of the TFC membrane compared to CTA. A real saline water sample collected from Hurgada, Egypt, with totally dissolved solids of 42,643 mg/L with NaCl as the draw solution (DS) at 25 °C and flow rate 1.55 L/min, was used to demonstrate the high performance of the prepared TFC membrane. The chemical analysis of desalted permeated water sample revealed the high performance of the prepared TFC membrane. Consequently, the prepared low-cost forward osmosis (FO) thin-film composite CTA membranes can be introduced in the desalination industry to overcome the high cost of reverse osmosis membrane usage in water desalination.

## 1. Introduction

The crisis of global water demands necessitates innovative and novel technologies that provide not only elevated throughput and productivity but also enclose optimum energy efficiency. Today, the most compatible and effective technology to produce fresh water from seawater is desalination using the reverse osmosis (RO) process. However, the high energy consumption and membrane fouling are the main challenges for large-scale application.

The spontaneous seepage of a solvent across a semipermeable barrier is referred to as forward osmosis (FO) [1,2]. Thermodynamically, FO process itself is spontaneous and requires no energy (if disregarding the energy for circulation) [3]. In general, during forward osmosis desalination processes, water flows from the feed solution (FS) to the draw solution (DS) where the feed solution becomes concentrated and the DS becomes diluted [4]; then product water is then separated from the diluted draw solution in the simple thermal distillation column recovery system for bench scale FO unit, but in hydride FO plant, the DS recovery system may be thermal distillation or RO unit according to the type of DS and the capacity of the FO plant. As a result, when FO is combined with a renewable energy DS recovery system, such energies would only be used for solute recovery, as FO benefits from being largely unpressurised, resulting in significant energy savings for FO desalination systems when compared to mainstream technologies such as RO [5].

FO processes have demonstrated benefits in terms of low fouling potential, ease of cleaning, bearing to high salinity, making them suitable for the treatment of high total dissolved solids (TDS) feed streams, and high-fouling load, such like landfill leachate, pharmaceutical, oil and gas produced water [6,7].

Cellulose acetate (CA) as well as cellulose triacetate (CTA) are the most widely utilized commercial membranes for FO applications. Due to its permeability and high hydrophilicity, CTA can be utilized in FO membrane fabrication. On the contrary, intensive reverse solute flux may hinder its extensive usage. Accordingly, the development of CTA-based FO membranes can be attained through the reduction of reverse solute flow [6]. Many articles have been reported on the preparation and performance of FO membranes such as cellulose acetate (CA) FO flat-sheet membranes [8], and the hollow fiber type of the CA membrane [9] revealed an extremely high solute reverse flux. To improve the performance of the FO membrane, various additives such as lactic acid, malic acid, and zinc chloride have been used as pore-forming agents [10]. Several hydrophilic nano-fillers, including aluminum oxide (Al_2_O_3_), carbon nanotubes (CNTs), silica (SiO_2_), zeolite, zinc oxide (ZnO), have been used to improve the performance of membranes in recent decades [11]. Because of its mechanical and electrical properties, GO has recently received significant attention in membrane enhancement. Because of the many reactive groups (e.g., OH, COOH) that enhance covalent or ionic interactions between the GO and the polymer matrix [12]. More recently, attention has been directed toward thin-film composite FO membranes due to the superior efficiency and broad pH implemented range. TFC-FO membranes are composed of two layers: an ultrathin layer above a porous substrate due to their high mechanical strength, satisfactory salt rejection, and hydrolytic stability [13,14]. Recently, phase inversion (a controlled polymer transformation technique from a liquid to solid phase) [15], interfacial polymerization (backed to two or more monomers that grow as polymer at the surface between two immiscible phases) [16,17], and layer-by-layer deposition (the film formation by deposition of oppositely charged layers) [17] strategies have been used to fabricate TFC–FO membranes.

The objective of this study is the preparation of low cost and energy-saving FO membranes for water desalination by modification of CTA by GO where both CTA and GO were prepared from palm fronds agro-waste that considered a renewable and abundant agricultural waste in Egypt, making it a potentially low-cost source of cellulose CTA-GO membrane was prepared via the incorporation of graphene oxide in membranes. TFC polyamide/CTA-GO was prepared via the interfacial polymerization method to improve the morphology, hydrophilicity, and FO efficiency as well as antifouling, consequently, enhancing the FO performance. To verify the high performance of the TFC-CTA modified membrane for desalination of saline water, a real feed water sample has been collected from Hurgada–Red Sea coast of Egypt with salinity 42,643 mg/L with NaCl as the draw solution (DS) at ambient temperature and a flow rate 1.55 L/min

## 2. Experimental

### 2.1. Materials

Toluene, ethanol, sodium hydroxide, sodium hypochlorite, potassium hydroxide, methylene chloride, glacial acetic acid, H_2_SO_4_, chloroform, NaNO_3_, H_3_PO_4_, KMnO_4,_ H_2_O_2_, HCl, and Dimethylformamide (DMF) were delivered via Sigma Aldrich. For the interfacial polymerization (IP) procedure, very high purity (>99%) of m-phenylenediamine, trimesoyl chloride, and n-hexane were utilized, and >99.5% NaCl as draw solute was used.

### 2.2. Preparation of Cellulose Triacetate from Palm Fronds

Palm frond wastes were obtained from the Siwa oasis, dried, milled into powder and dewaxed in a mixture of toluene and ethanol overnight, and the crude cellulose was extracted by using sodium hydroxide. The crude cellulose was bleached using sodium hypochlorite. The bleached cellulose was treated with potassium hydroxide to extract α-Cellulose, and lastly, acetylation of cellulose was conducted by dissolving in methylene chloride, acetic anhydride, glacial acetic acid, H_2_SO_4_ in accordance with our prior work [18,19].

### 2.3. Synthesis of Graphene Oxide (GO)

GO was prepared from palm fronds according to the modified Hummer method [20]. Palm fronds wastes were heated at 650 °C. 5 g from prepared graphite and 2.5 g NaNO_3_ was mixed with 108 mL H_2_SO_4_ and 12 mL H_3_PO_4_ in an ice bath for 10 min. Then, 15 g of KMnO_4_ was added gradually while keeping the temperature of the mixture below 5 °C. The suspension was stirred for 60 min in an ice bath after reacting for 2 h in an ice bath. It was then stirred for another 60 min in a 40 °C water bath. The temperature of the mixture was kept constant at 98 °C for 60 min while the water was continuously added. Deionized water was then added until the suspension had a volume of 400 mL. After 5 min, 15 mL of H_2_O_2_ was added. The reaction product was centrifuged and washed repeatedly with deionized water and a 5% HCl solution. Finally, the graphene oxide was dried at a temperature of 60 °C.

### 2.4. CTA Fabricated Membrane

A phase inversion technique was introduced for FO fabrication. Dried CTA prepared from palm fronds was added to a container containing dimethylformamide (DMF) using weight ratio20 polymer: 80 solvent weight ratio and was stirred using a mechanical stirrer. Preceding to the membrane casting, the casting glass sheet was cleaned with tap water and acetone then dried before use. The mixture was stirred at 30 °C until a homogeneous solution was obtained. To eliminate air bubbles, the polymer mixture was placed at 30 °C in the oven for an hour. The polymer solution was cast onto a dry-clean glass sheet using 200µm casting knife. After 45 s for partial solvent evaporation, the glass sheet was immersed into a water coagulation bath at 20 °C. The as-cast membrane was detached from the glass plate and submerged in a tap water bath. CTA membranes were annealed in a hot DI water bath at 75 °C for 2 min. 24 h drenching in deionized water is introduced to the solidified membranes before the performance test [21].

### 2.5. CTA/GO Membrane Preparation

GO was introduced to DMF solvent and then ultra-sonication for 30 min before dissolving CTA in the blended solution. The mixture was stirred at 30 °C until a homogeneous solution was obtained. Then the solution was kept in an oven at 30 °C for several hours to tear out the air bubbles. The compartments of the cast solution, according to the previously reported [21,22], are listed in Table 1.

Membrane performance can be influenced by three major parameters: polymer composition, solvent type in the polymer solution mixture, and demixing rate during the coagulation bath. The type of demixing that occurs during phase inversion (PI) using DMF, which has been reported to be quick, results in drop-shaped voids. DMF has a high polymer solubility parameter. The DMF-based casting mixture precipitates in a very short period of time [23].

The coagulation bath temperature, on the other hand, is an important factor in determining membrane properties that impacts the phase inversion thermodynamics and membrane structure. The bath temperature affects the rate of the solvent–non-solvent exchange and the movement of the polymer chains. Thermodynamically, a higher temperature results in faster movement of the polymer chains, improving the rearrangement of the polymer chain at the skin layer before the CTA chains are fixed by solidification. Presently, the cast film was immersed in water at 20 °C since higher bath temperatures are reported to lower water permeability of the resultant membranes [21].

### 2.6. Preparation of Polyamide Thin Film Composite on CTA/GO Support

The interfacial polymerization (IP) technique was used for the preparation of the polyamide (PA) active layer on a CTA/0.3%GO substrate using MPD and TMC monomers. First, hydrolysis of the cellulose triacetate membranes in NaOH solution (0.7 wt%, 30 min) was undertaken, fabricating more permeable membranes for both water and salt [24].The substrate was sunken in a 2.0 wt% MDP aqueous solution for a minute then the extra used MPD solution was taken away, then, 0.1 wt% TMC solution in *n*-hexane was introduced to the above-soaked substrate for 1 min. To remove the residual monomers TFC membrane was rinsed with DI water [25].

### 2.7. The Fabricated Membrane’s Performance

**Figure 1:**
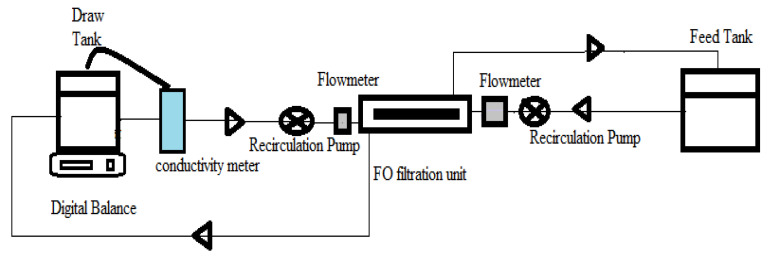
The schematic diagram for bench scale forward osmosis (FO) unit.

The efficiency of the fabricated membrane was examined through an in-house designed FO lab-scale unit (Figure 1), where DI water was used as the feed solution and 2 M NaCl was used as the draw solution. The product water was then separated from the diluted draw solution in the simple thermal distillation column recovery system.

The permeation properties of the prepared membranes were evaluated using a cross-flow filtration unit at a trans-membrane pressure (TMP) of 1.55 MPa and 1 L/min feed flow rate. The water flux (Jw) (Equation (1)) of the membrane was gained by calculating the water’s volume (ΔV) passing through the active and efficient filtration area (Am) of the membrane through the collection time of the sample (Δ t), the effective filtration area of all the membranes was 56.3 cm^2^.

(1) $$\mathrm{Jw}=\frac{\Delta V\;}{\Delta \;t\;*\mathrm{Am}}$$

A (water permeability) was calculated by

(2) $$A=\frac{\mathrm{Jw}}{\Delta \;P}.$$

The salt permeability coefficient (B) was calculated at 1.55 MPa and 2 M NaCl solution by Equation (3);

(3) $$B=\mathrm{Jw}(\left(\frac{1}{R}-1\right))$$

The apparent salt rejection (R) was calculated using Equation (4)

(4) $$R\%=\left(1-\frac{\mathrm{CNaCl},\mathrm{ds}\;\times \mathrm{Vds}}{\mathrm{Cfs}0\times \mathrm{Vfs}0}\right)\times 100.$$

Using pure DI H_2_O as an FS, the reverse DS flux was estimated by Equation (5)

(5) $$\mathrm{Js}=\frac{C_{f}V_{f}-C_{0}V_{0}}{A\Delta t}$$

where Js is the reverse salt flux (g/m^2^·h), C_0_ is the initial feed concentration expressed in g/L and C_f_ refers to the final feed concentration (g/L), V_0_ and V_f_ refers to the initial and final volumes of the feed solution (L), respectively, Δt is the time (h), and A is the active and efficient filtration area membrane surface area (m^2^) [26].

### 2.8. Characterization of Prepared Materials

The prepared CTA and GO were fully characterized by XRD measurements using (D8 Advance, Bruker, Germany) diffractometer equipped with a position-sensitive detector Lynxeye (Cu*K*α = 1.54 Å). FTIR (6100 Jasco., Tokyo, Japan) Spectrum equipment with 4 cm^−1^ resolution and frequency range of 400–4000 cm^−1^ was utilized for recording the FTIR spectra of samples. An SEM (S3400N, Hitachi, Japan) was utilized to determine the morphology of samples. Nuclear magnetic resonance (NMR) was determined using an Avance III 400 MHz Bruker High-performance digital, FT-NMR spectrometer. The prepared membranes were also fully characterized by FT-IR, porosity, the water contact angle measurement as well as morphological and mechanical properties of the prepared membranes. A tensile test was performed using the H5KS universal tensile measuring unit in which the space between the handles was set to 200 mm and the thickness of the membranes measured was 25 mm. Samples were stretched at a steady elongation rate of 30 mm/min using a collection of five samples to obtain the average reading.

## 3. Results and Discussions

### 3.1. Material’s Characterization

#### 3.1.1. Characterization of CTA

**Figure 2:**
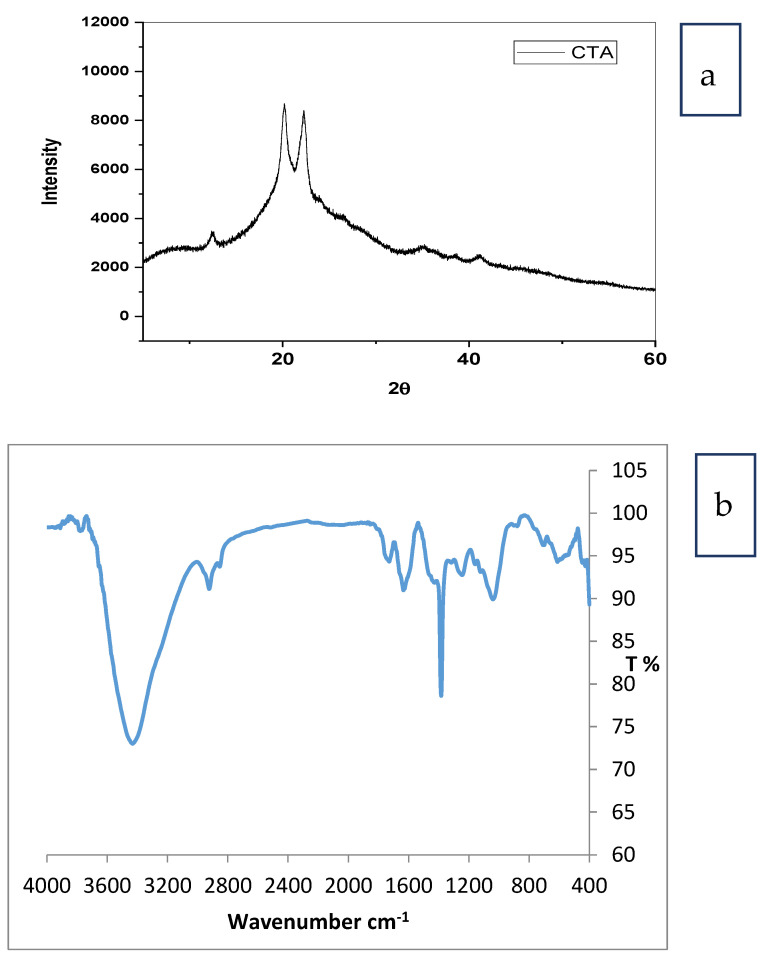
(**a**) XRD and (**b**) FTIR spectrum for cellulose (triacetate) prepared from palm fronds.

Figure 2a shows the XRD pattern of cellulose triacetate. The peaks detected at 12.27, 15.8, 20.6, and 33.7 were characteristic of Iβ phases of cellulose [27,28,29,30]. The peak detected at 8.7 was characteristic of the semicrystalline acetylated derivative cellulose. The substitution of a hydroxyl group by the acetyl groups breaks the inter and intramolecular hydrogen bond of cellulose which lead to a decrease in the degree of the crystallinity [31].

Figure 2b shows the FTIR spectrum for cellulose triacetate. The peak at 1730 cm^−1^ was characteristic to wide CO band for bonding acetyl groups. A small band at 1369 cm^−1^ was assigned to C–H bond in OCOCH_3_ group and the band detected at 1240 cm^−1^ was assigned to CO stretching of acetyl groups [32]. An intensive band at 1051 cm^−1^ was assigned to skeletal vibration from the C O C pyranose ring. There are no bands detected for the unreacted acetic anhydride and the by-product, acetic acid [33]. The peaks at 1050 and 1390 cm^−1^ backed to C–O alcoholic stretch and C–H from cellulose and hemicelluloses, respectively [34].

**Figure 3:**
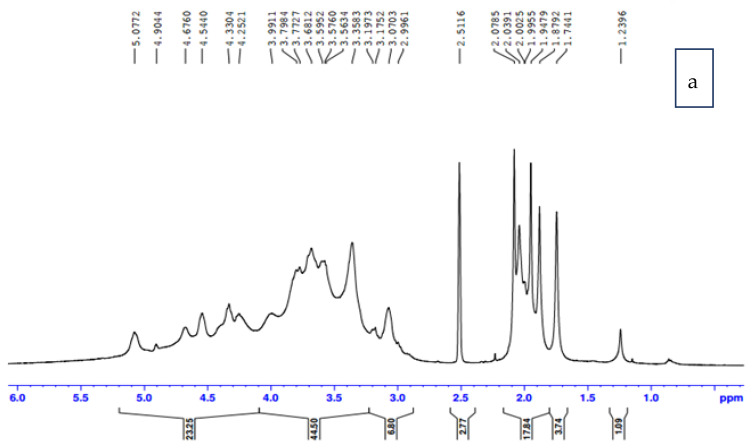

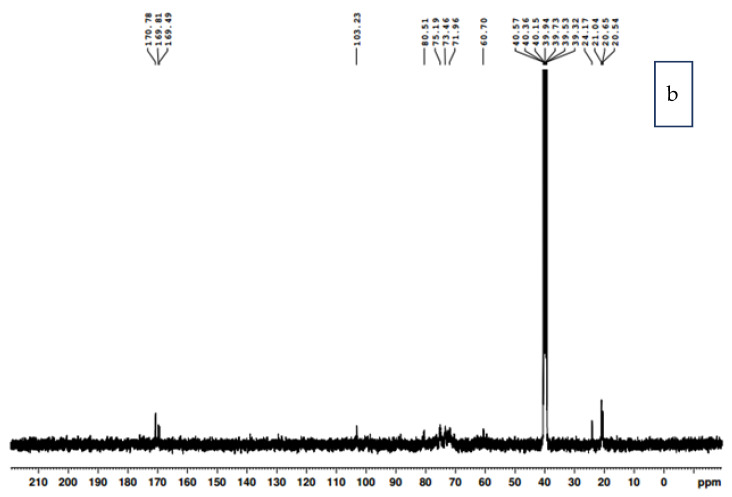
(**a**) ^1^H NMR spectrum of chloroform soluble CA palm fronds (**b**) ^13^C NMR spectrum of chloroform soluble CA palm fronds.

NMR spectroscopy is an elucidation tool for CTA structure definition. NMR proves the formation of CTA through acetylating CA. Figure 3a shows the proton NMR as an indicator for the degree of substitution (DS). The ratio of the seven anhydrocellulose proton absorbance was utilized in the specific range between 3.6 mg/L and 5.1 mg/L to the absorbance of three methylated protons of acetyl group between 1.8 mg/L and 2.1 mg/L. DS resulted from dividing 1/3 (acetyl peak area) by 1/7 (anhydroglucose area). DS Acetyl resulted from the ratio of spectral integrals of acetyl moiety to the repeating unit. The DS of crude cellulose acetate was 2.83 and soluble chloroform3.17. Accordingly, it was proven that the average DS was nearly the same at the individual 2, 3, and 6 hydroxyl positions of the glycosyl ring (DS_2_, DS_3_, and DS_6_).

^13^C-NMR showed significant signals appearing at δ value170.7 referring to carbonyl carbon plus that at δ value20.5, δ20.6, 21.0, and δ21.5 set for methyl carbons, which was not detected in the cellulose spectrum confirming the supposed form of acetyl cellulose structure, as shown in Figure 3b. In addition, carbons attached to the acetyl groups became slightly shifted down field than the parent structure. In addition, the amount of substituted carbon was identified by ^13^C spectrum. Chloroform soluble cellulose acetate spectrum indicated the highest number of carbons, i.e.,^13^C spectrum of chloroform soluble cellulose acetate revealed the presence of 24 carbons.

#### 3.1.2. Characterization of Graphene Oxide

**Figure 4:**
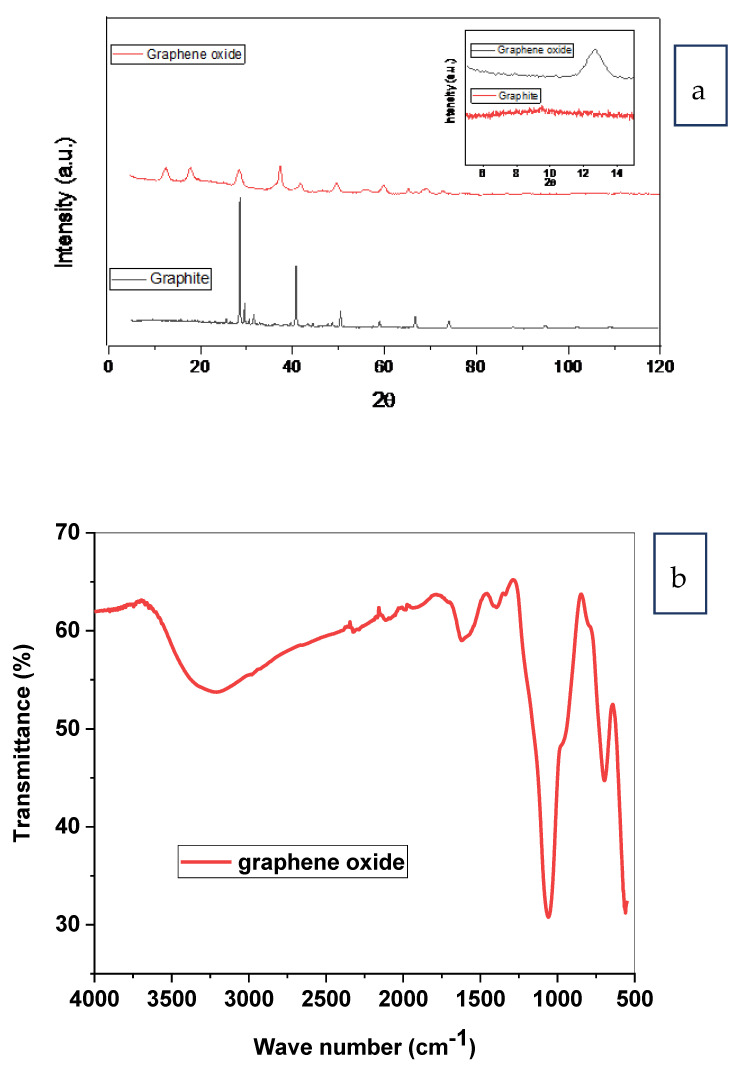
(**a**) XRD patterns of graphite and graphene oxide. The inset shows the characteristic graphene oxide extension peak and (**b**) FT-IR spectrum of graphene oxide.

Graphene oxide was obtained from palm fronds through graphite by hammer technique [35]. Figure 4a showed the XRD pattern of graphene oxide. The diffraction peak at 2 θ = 12.68°correspondedto graphene oxide [35], which was in agreement with that detected at 2 θ = 11.6° for graphene oxide prepared from agricultural sugarcane bagasse oxidation [36]. Decreasing the intensity of the native graphite peak, between 2 θ of 25° and 30°, revealed the oxidation of the starting graphite to graphene oxide [37].

The band at 3000–3650 cm^−1^ in Figure 4b was attributed to the extended pulses of OH groups of H_2_O molecules adsorbed on GO. The peak of 1623 cm^−1^ was assigned to the stretching vibration of C=C, while the peak of 700 cm^−1^ stretching vibration of C=O of carboxylic acid and carbonyl groups presented at the boundaries of graphene oxide. The peak of 1390 cm^−1^ was attributed to the extended pulses of C–O of carboxylic acid. Meanwhile, the peak of 1063 cm^−1^ was attributed to the extended pulses C–OH of alcohol [35].

#### 3.1.3. Characterization of Prepared CTA and CTA/GO Membrane

##### Contact Angle Prepared CTA and CTA/GO Membrane

The contact angle values show that the CTA/GO membranes become more hydrophilic as GO- was incorporated into the cellulose triacetate membrane matrix. The water contact angle diminished from 65° (pure CTA membrane) to 47° as the amount of GO was increased to 0.3 wt% GO. The water-loving membrane could drop off the immediate interplay between the adsorbed thin layer of water molecules on the top of the membrane [38]. The GO enhanced the membrane’s surface negativity due to the existence of oxygen-containing functional groups such as hydroxyl, epoxy groups, and carboxyl groups in GO which increase the interaction of membrane and water molecules, resulting in increased hydrophilicity.

A significant decrease in contact angle after the addition of 0.3 wt% GO content was observed, demonstrating a significant increase of surface hydrophilicity which is envisaged to confer greater FO flux performance. This is supposed to be related to hydrophilic GO movement toward the top layer during phase inversion, leading to the lower water contact angles because of the GO’s superior attraction of OH groups, however, the contact angle of 0.5 wt% GO was 54. This might be attributed to the bad dispersion of GO onto the surface of the membrane surface, as well as the bad compatibility and adhesive forces between the GO and the polymer, as the filler content was increased.

##### SEM Prepared CTA and CTA/GO Membrane

**Figure 5:**
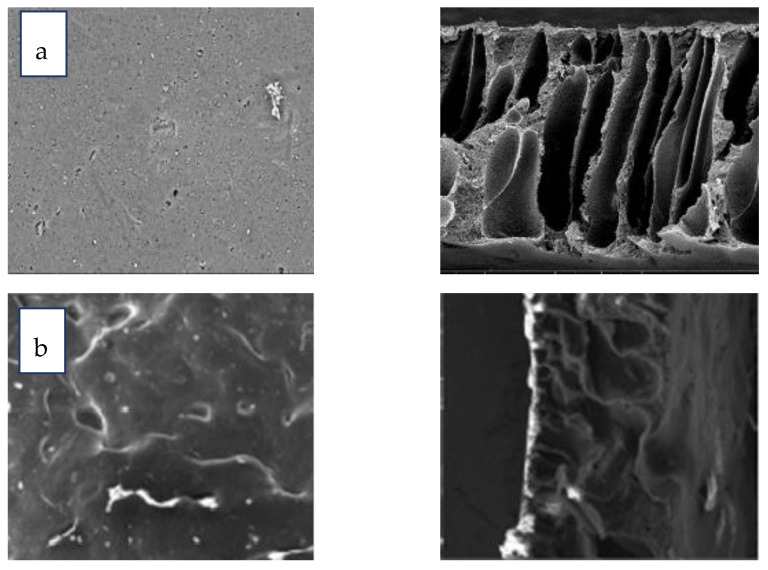

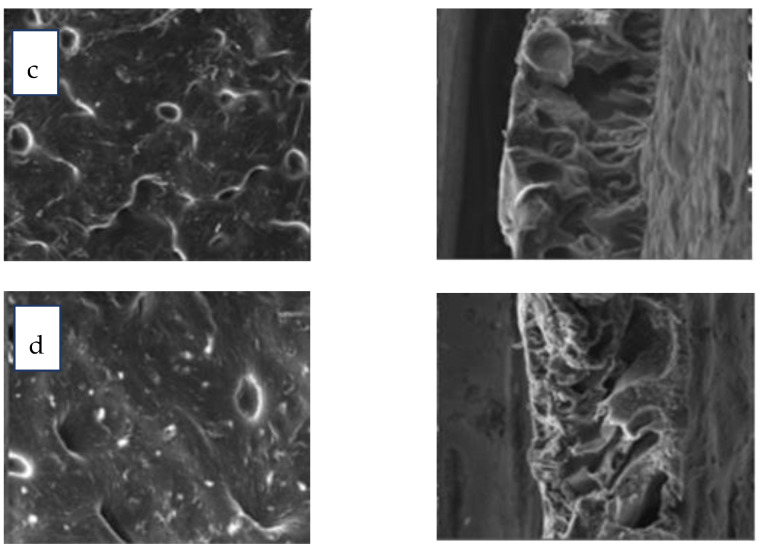
SEM images of the surface (magnification 800) and corresponding cross-section (magnification 2000) (**a**) Pure CTA, (**b**) 0.1 wt% wt% CA/GO, (**c**) 0.3 wt% wt% CA/GO, and (**d**) 0.5 wt% wt% CA/GO.

The morphological structures of the CTA and CTA-GO (0.1, 0.3, and 0.5 wt%) membranes were evaluated through SEM (Figure 5). Pure CTA membranes showed the existence of both sponge and a few macrovoid structures in the cross-section area, which is distinguishing of corresponding membranes [39,40]. The increase of these macrovoid structures on the addition of GO to CTA-GO membranes seems to be larger in comparison to pure CTA membranes (Figure 5).

CTA-GO membranes are endowed with an appreciable pore size compared to that of pure CTA membranes, which give rise to high water permeability. The surface of the CA membrane, after the adding of GO, shows well distribution of GO particles onto the polymer matrix. Moreover, no cracks were observed on the membrane’s surface on the addition of GO indicating the GO membrane’s strength.

From the SEM analysis, the cross-section area decreased in thickness after the addition of 0.1 wt% GO and could be due to hydrophilic GO particles, which can increase the rate of water (non-solvent) and solvent mixing during phase inversion, leading to a relatively lean top surface [41]. The thickness descends due to the incorporation of GO, which is aligned with previous studies [42]. Furthermore, an increase in the number of GO particles augmented the viscosity of the dope solution leading to a diminished mass transfer rate of the non-solvent, giving rise to the thickness of the skin layer. When the quantity of GO on the CTA matrix was raised to 0.5 wt%, macrovoids reduced and the connectivity of the holes was subdued, which was attributed to the increase in viscosity ascribed to the GO (Figure 5d).

##### The Porosity of Prepared CTA/GO Membrane

**Figure 6:**
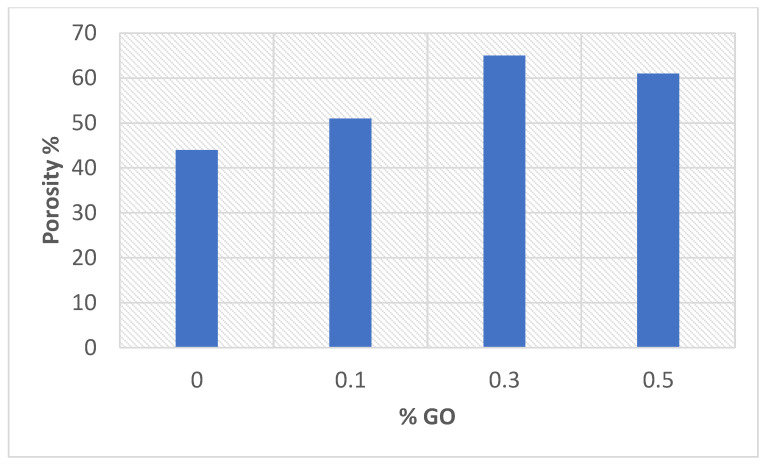
The % porosity as function of % GO incorporated in the prepared membrane.

It is clear that the percentage porosity increased from 44 to 56% as the %GO increased to 0.5% compared with CTA membrane (Figure 6). As a result of the GO presence in the casting solution, the porosity of the CTA/GO membrane increased; extra pores developed on the membrane surface. OH and COOH of GO show the hydrophilic characteristics of polymer matrix and may improve the membrane synthesis process by boosting the rate of solvent/non-solvent exchange via the inversion technique [43]. The addition of GO in the casting solution increases hydrophilicity due to the strong attraction of water molecules by GO and the inclusion of hydrophilic OH and COOH groups on the active membrane substrate (AL). It is possible to deduce that the greater the porosity, the greater the permeability and the water flux.

##### Mechanical Properties of Prepared CTA/GO Membrane

**Figure 7:**
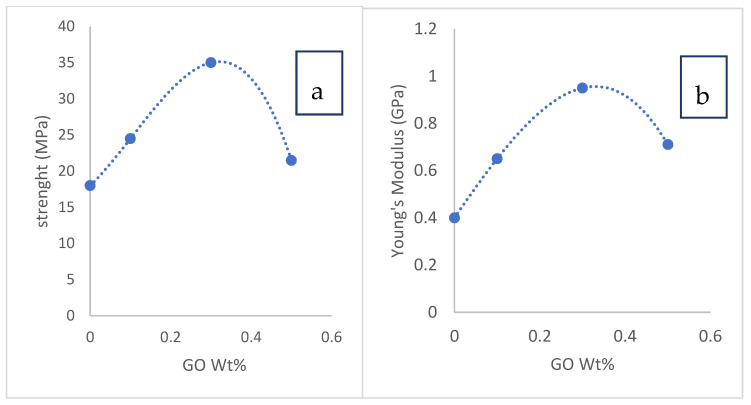
(**a**) Membrane strength versus GO concentration and (**b**) and Young’s modulus for the CTA/GO membranes with increasing GO loading.

The mechanical assessment is one of the most important considerations for determining a component’s load-bearing capability. Mechanical properties are required for the practical uses of a manufactured porous membrane. The vigor of the prepared CTA/GO membranes was scanned and the findings represented in Figure 7a; the strength was 18 MPa for the pure CTA membrane. By increasing the GO concentration, an increase in the membrane strength up to 35 MPa at 0.3 wt% GO loading was observed; further GO loading reduced the strength to 21.5 MPa.

Figure 7b displays the Young’s modulus of the CTA/GO membrane, indicating a similar behavior to the variations in strength. The existence of 0.3 wt% of GO resulted in an optimal increase in Young’s modulus (0.95 GPa) compared to the CTA membrane (0.4 GPa). The incorporation of GO enhanced the strength and Young’s membrane modulus for the fabricated CTA/GO membranes. The reinforcement effect was due to the efficient transition of the mechanical characteristics of the graphene, which was also related to the highly interfacial adhesion between the GO and the CTA chains. Furthermore, GO’s wrinkled surface texture may induce mechanical overlapping and intensify adhesion to the polymer matrix [44,45]. It was desirable to increase the performance of the graphene reinforcement of the CTA membrane. At higher GO loads of more than 0.3 wt% percent, the strength, as well as Young’s modulus, decreased. This could result from a reduction in the dispersion quality of GO in the matrix [45]. Therefore, the optimum strength and Young’s modulus were observed at 0.3 wt% GO, which was envisaged to fulfill the usage requirements.

##### The Parameters of Fabricated Membranes

The permeation properties and membrane parameters of fabricated membranes is presented in Table 2. The experimental results demonstrated that the CTA/0.3 wt% GO membranes provided higher water flux and comparable salt rejection percentage due to the attained small B/A ratio and Js/Jw ratio [46,47,48,49].

#### 3.1.4. The Performance of Fabricated CTA/GO Membranes

The enhancement in hydrophilicity of the membranes was indicated by the decrease in the water contact angle of the CTA/GO membranes from 65° (pure CTA membrane) to 47°as GO content was increased to 0.3 wt% GO. As a consequence, the water permeation was improved. It was, however, observed that in the case of CTA/0.5 wt% GO membrane, the water flux of the membrane decreased, becoming even less than that of the pure CTA membrane. This can be attributed to the use of graphene particles in the membrane matrix as a hindrance to water flow. So, the average route was increased, which led to a reduction in the permeate flow. In general, water flux was influenced by the hydrophilicity as well as the hindrance effect of GO. Firstly, hydrophilicity may be the predominant force in comparatively low GO loads (up to 0.3 wt%), then it was a hindrance to GO in higher loads. Secondly, the viscosity of the casting solution increased at with extra GO dose. It could restrict the existence of large pores, which were easy to transport through water [50]. The increased viscosity also contributed to a decline in the dispersion efficiency of GO [46,47].

The reverse salt flux inversely proportioned to GO concentration Up to 0.3 percent wt. When GO loading rose further, the reduction in reverse salt flow slowed down as a result of the hindrance effect of GO in the membrane. The J_s_ of the hybrid membranes was fairly low, demonstrating a moderate salt rejection of the membranes [43,44]. The Js/Jw (specific salt flux) of CTA/0.3 wt% GO was 0.06 g/L. Consequently, the CTA/0.3 wt% GO membrane has good water permeation and low reverse salt flux compared to CTA membrane.

##### The Effect of NaCl Concentration as a Draw Solution

**Figure 8:**
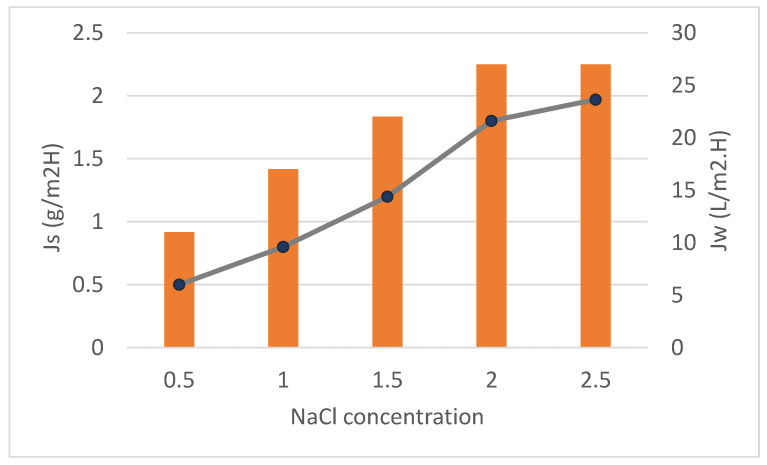
The effect of NaCl concentration as a draw solution using CTA/0.3% GO under constant operating conditions.

Figure 8 shows the effect of different concentrations of NaCl as draw solutions (0.5 and 2.5 M) with feed solutions of DI on the water flux as well as the RSF of neat CTA and CTA/GO membrane at a constant flow rate (1.55 L/min), temperature (25 °C) and the effective membrane area of (56.3 cm^2^). Interestingly, it was detected that the water fluxes increased from 11 to 27 L/m^2^h whilst the RSF increased from 0.5 to 1.97 g/m^2^h for CTA/0.3% GO. This might be attributed to increasing the osmotic driving force. This was owed to the enhancement of the net driving strength via the membrane for both water and NaCl at a high DS content [51]. Comparing the RSF for CTA/0.3% GO membrane with the neat CTA membrane at different DS concentrations resulted in slightly higher RSF for CTA/0.3% GO membrane than the neat CTA membrane. This might be due to the salt concentration gradient growth over the membrane sides that provide a higher driving force for the migration of NaCl ions from the DS side to the FS side [52].

##### Membrane Orientation and Its Impact on the Performance of the Fabricated Membranes

**Figure 9:**
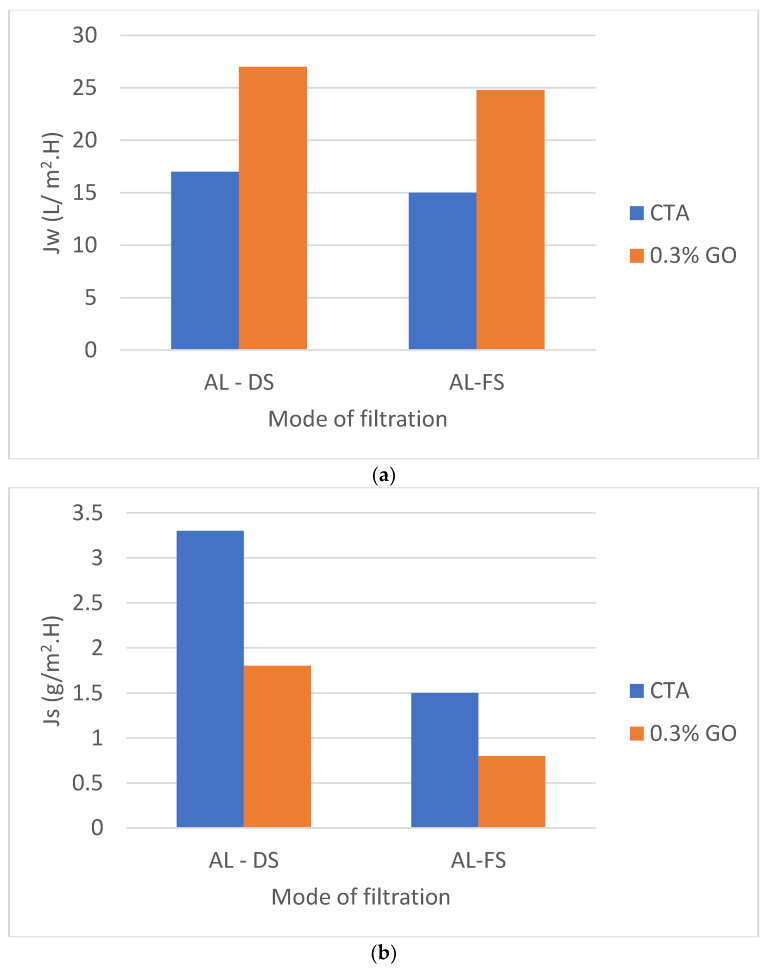
The effect of membrane orientation on (**a**) the water flux of and (**b**) on the reverse salt flux of CTA/0.3% GO compared with CTA membrane.

Two modes can be tested by facing the active layer either to the feed solution (LA-FS) or the draw solution (AL-DS) utilizing DI and 2 M NaCl as FS and DS, respectively, with a 1.55 L/min flow rate. The CTA and CTA/0.3% GO membranes flow rate at AL-DS orientation were slightly higher with respect to AL-FS orientation (Figure 9a) as a result of the disappearance of internal concentration polarization (ICP) by utilizing the DI water as FSs on the SL part of the prepared membrane [53,54].

Water flowing from the feed tank via the membrane SL dilutes DS at the membrane surface, diminishing the osmotic pressure on the membrane surface and, as a result, limiting the water flow [6].

Dilutive ICP has a significant impact on water flux in the FO process because the osmotic pressure of the DS at the membrane boundary layer is critical to the FO process. Concentrative external concentration polarization (ECP) is missing on the AL side of the membrane when DI is used as the FS in the FO style.

The occurrence of the ICP is inverted when the system is set up in PRO mode. Because the PRO-style DS is facing the AL membrane, the effect is dilutive ECP, which can be reduced by delivering cross-flow shear to the membrane superficially. Because the concentrative ICP resides on the feed part of the membrane, its influence is less important than that of the dilutive ICP, which explains why the water flow in the PRO mode is greater than that in the FO mode [11].

The RSF (Js) of CTA membrane were 1.5 and 3.3 g/m^2^h in the AL-FS and AL-DS types, respectively and for CTA/0.3 percent GO membrane were 0.8 and 1.8 g/m^2^h in the FS-DS direction and AL-DS direction sequentially (Figure 9a). The solute concentration of the membrane surface is higher in the PRO process mode than in the FO mode operation, resulting in higher driving power and higher water flow. Higher draw concentration at the membrane surface, on the other hand, typically resulted in higher RSF in the PRO style [48].

### 3.2. Formation of Polyamide Thin Film on CTA/0.3% GO Support

TMC (Trimesoyl chloride) was used as a linking molecule to successfully synthesize polyamide (PA) on the cellulose triacetate (CTA) substrate. The PA coating on the CTA/0.3 percent GO membranes resulted in somewhat yellowish membranes.

**Figure 10:**
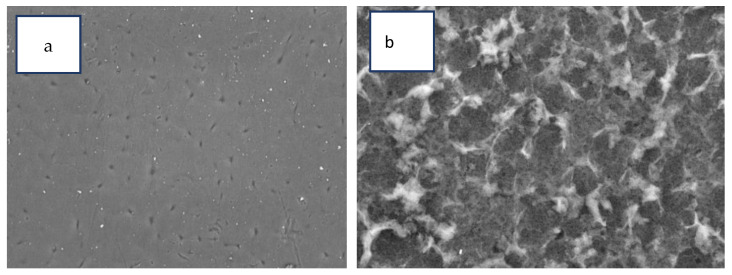
SEM images (magnification 4000) of (**a**) CTA/0.3%GO substrates and (**b**) the PA-TFC membrane.

SEM micrographs clearly display the traditional “ridge and valley” configuration of PA (Figure 10b). However, there are clear distinctions between the neat CTA/0.3 percent GO displayed a smoother thick surface morphology (Figure 10a) with a few small pores structure and TFC membranes had rougher, grass-like structures. [24,25,26]. There were also more open top surface structures found.

**Figure 11:**
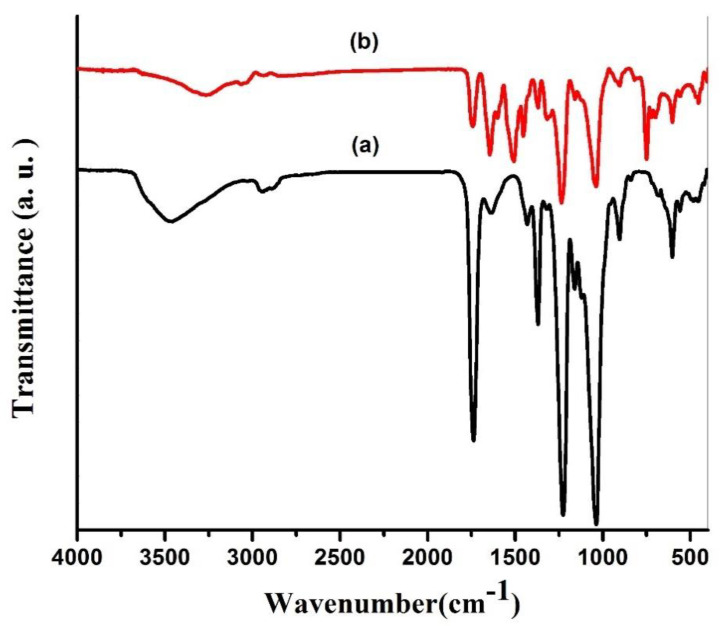
ATR-FTIR spectra of (**a**) CTA/0.3% GO substrate and (**b**) the PA-TFC membrane.

The FI-IR spectra in Figure 11 indicate that distinctive CTA peaks were recorded at 1221 cm^−1^, 1369 cm^−1^, and 1720 cm^−1^, which were aligned with the expansion vibrations of C-O-C, C-CH_3_, and C=O, respectively. Significant peaks at 3430 and 2939 cm^−1^ were correlated with the stretching vibration of the O-H and C-H bands, respectively, with higher GO loading (0.3%) [55]. The presence of two significant peaks at 1661 cm^−1^ and 1541 cm^−1^ only occur on the top TFC surface membrane after IP reaction reflecting the C=O expansion band (amide I) and C-N expansion band (amide II) of PA, consequently, confirms the effective development of PA thin film [56].

#### 3.2.1. The Performance of Fabricated TFC Membranes

TFC-FO membrane with CTA/0.3% GO substrates shows a high A value, revealing their superior water permeability and low salt permeability coefficient (B) [25] and lower (B/A) ratio (Table 3).

**Figure 12:**
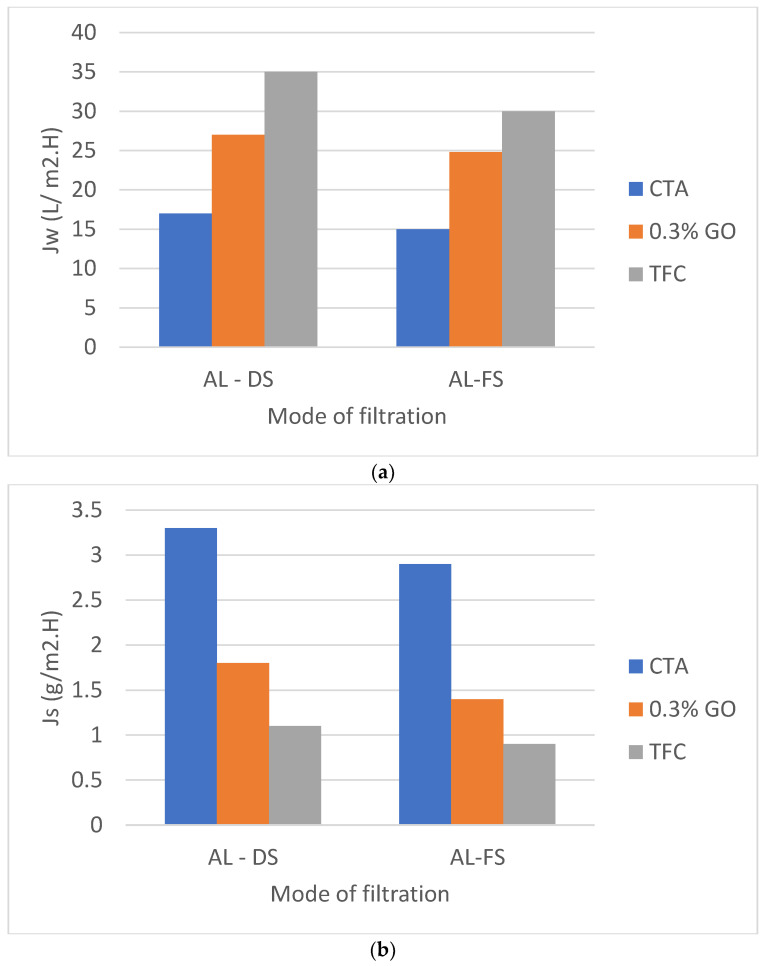
The effect of membrane orientation on (**a**) the water flux and (**b**) the reverse salt flux of TFC (CTA/0.3% GO) substrate compared with CTA/0.3% GO and pure CTA membrane.

Figure 12 a indicates that the water flux Jw of TFC (CTA/0.3% GO) was 35 L/m^2^h in AL-DS mode, 30 L/m^2^h in AL-FS mode) are much higher than those of pure CTA (17 L/m^2^h in AL-DS mode, 15 L/m^2^h in AL-FS mode) and CTA/0.3% GO (27 L/m^2^h in AL-DS mode, 24.8 L/m^2^h in AL-FS mode) which indicates the enhancement of the water flux for prepared TFC membrane. Meanwhile, the reverse salt fluxes of the TFC (CTA/0.3% GO) was 1.1 g/m^2^h in AL-DS mode, 0.9 g/m^2^h in AL-FS mode) are much lower than those of pure CTA (3.3 g/m^2^h in AL-DS mode, 2.9 g/m^2^h in AL-FS mode) and CTA/0.3% GO (1.8 g/m^2^h in AL-DS mode, 1.4 g/m^2^h in AL-FS mode) (Figure 12b). Therefore, the specific salt flux, Js/Jw of TFC (CTA/0.3% GO) substrate membrane was 0.03 g/L, telling that the TFC membrane held both good permeation for water as well as low reverse salt flux compared to CTA and CTA/0.3% GO membrane (Table 3).

The enhancement in water flux of TFC with respect to CTA/GO membrane may be due to higher water sorption properties and swelling/deswelling effect of the PA layer. According to Donnan theory, there is an exchange of mobile ions (OH^−^, Na^+^, H^+^ or Cl^−^) between the macromolecule (PA layer) and the solution. This swelling/deswelling effect enhances the permeation properties of TFC as the result offast diffusion of water molecules across the membranes [57], as shown in Table 3 (a high A value, revealing their superior water permeability and low salt permeability coefficient (B) [58].

It is difficult to conduct a fair comparison between CA, CTA, and TFC-FO studies since they used different polymer weight percent, solvents, GO percent, and monomers for thin-film polymerizations and the type of DS used, however, Table 4 demonstrated the high performance of prepared CTA, CTA/GO, and TFC from palm frond, which was considered a challenge to prepare FO membrane from (CTA and GO) extracted from agriculture waste with superior performance than previously reported FO membranes prepared from commercial (CTA and GO).

#### 3.2.2. FO Fabricated Membranes and Its Operation in Sea Water Sample

The freshwater recovery from salty water is a favorable pressing issue confronting Egypt at present, as the country suffers not only a water deficit but also a succession of energy crises. The neat CTA/, CTA/0.3% GO, and polyamide TFC on CTA/0.3% GO substrates were used for desalination of a natural seawater sample taken from Egypt’s Hurghada-Red Sea coast utilizing NaCl as the DS.

Table 5, Table 6, Table 7 and Table 8 demonstrate the sample properties prior to the FO operation. The FS includes Red Sea collected water sample with 42,643 mg/L of total dissolved solid (TDS) and 2 M NaCl as DS, a flow rate of 1.55 L/min at 25 °C, and a membrane operation area of 56.3 cm^2^ in FS-AL mode.

**Figure 13:**
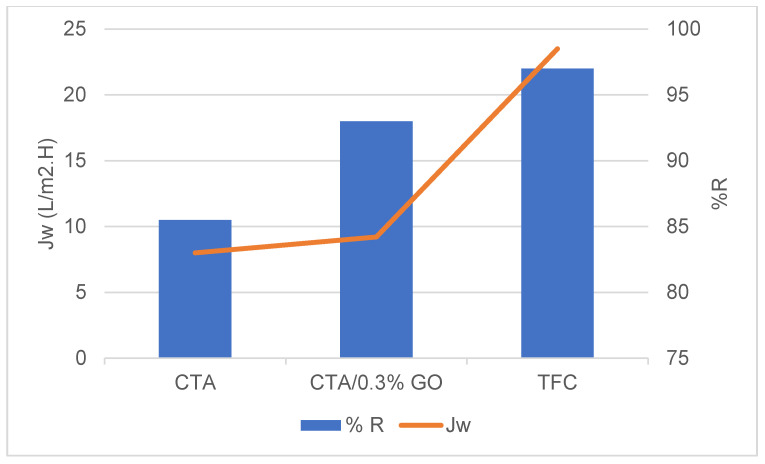
Water flux and salt rejection using a natural seawater sample collected from Hurgada–Red Sea coast of Egypt and NaCl as the draw solution (DS) at 25 °C, flowrate1.55 L/min, FO mode: with cells vertically oriented, and active layer—faced feed solution (AL-FS), using Neat CTA, CTA/0.3%GO, and TFC modified membranes.

The combination of high TDS and organics in the natural seawater sample resulted in the effective concentration of constituents that was greater than that in the synthetic saltwater replica samples Figure 13. Furthermore, the natural seawater sample contains a large amount of strong TDS ions Table 5, Table 6 and Table 7. Membrane fouling, including organic foulants as well as inorganic foulants, is expected to be a significant cause of the intense flux decrease seen in Figure 13. As a result, as DS/FS concentrations shift, both external concentration polarization (ECP) and internal concentration polarization (ICP) change. High FS concentricity also led to an increase in ECP, which directly resulted in a significant reduction in the resulting water flux due to a decrease in osmotic pressure through the membranes [22]. (R%) of the FS is assessed by collecting and reviewing the DS sample at the end of each test (Table 8). After 3 h, all desalted permeated water samples were obtained for chemical analysis. Table 9 and Table 10 reveal that TFC modified membranes have superior performance according to the quality of the produced permeate.

It is revealed that, for the FO process, TFC on CTA/0.3% GO substrate membrane led to better salt rejection than neat CTA and CTA/0.3% GO membranes. FO technique has many advantages, such as decreasing the amount of salt without using hydraulic pressure and it is considered an ecologically friendly technique where it not only saves energy but also it saves desalination cost. Table 9 and Table 10 compare the permeate water quality of TFC on CTA/0.3% GO against the Egyptian Ministerial decree No. 458/2007 for drinking water. The water showed that the permeate water meets the guidelines for drinking water.

## 4. Conclusions

CTA/GO membranes were synthesized by phase inversion using CTA and GO prepared from palm fronds. The GO% ranged from 0.1–0.5%. The GO incorporation into the CTA matrix led to an increase the membrane hydrophilicity, porosity, and mechanical strength. These increases resulted in significant improvement in water flux as compared to neat CTA. The water contact angle diminished from 65° (pure CTA membrane) to 47° as the amount of GO was increased to 0.3 wt% GO% porosity increased from 44 to 56% as the content of GO increased to 0.5 wt% compared with that of CTA membrane. The enhancement in the hydrophilicity and mechanical strength of the membrane due to the high attraction of water molecules by GO and the inclusion of hydrophilic reactive groups (OH and COOH) on the active membrane substrate (AL).

The specific salt flux, Js/Jw, of the TFC (CTA/0.3% GO) substrate membrane was 0.03 g/L which is lower than CTA and CTA/GO. As a result, the TFC membrane surpassed the CTA and CTA/0.3 percent GO membranes in terms of both water permeability and reverse salt flux. A real salty water sample from Egypt’s Red Sea coast was used as the feed, with the draw solution of NaCl at 25 °C and flow rate1.55 L/min. The quality of desalted permeate indicated that the high-performance polyamide TFC–CTA/0.3% GO. As a result, the prepared TFC–CTA showed a high potential for interest in saline water desalination. Consequently, the TFC membrane platform will most likely replace the CA membrane for FO, particularly when combined with a renewable energy DS recovery system, where such energies would only be used for solute recovery.

## Figures and Tables

**Table 1:** The compositions of the casting solution.

	Polymer%	Solvent%	
	CTA (wt %)	DMF %(wt %)	GO% (wt %)
M1	20	80	0
M2	19.9	80	0.1
M3	19.7	80	0.3
M4	19.5	80	0.5

**Table 2:** Permeation properties of fabricated membranes.

GOwt%	Jw (L/m^2^h)	Js (g/m^2^h)	Js/Jw g/L	A (Lm^−2^ h^−1^bar^−1^)	B (Lm^−2^h ^−1^)	B/A (bar)
0	17	3.3	0.19	10.96	1.93	0.17
0.1	22	4.6	0.21	14.19	2.64	0.18
0.3	27	1.8	0.06	17.41	2,765	0.16
0.5	25	1.8	0.07	16.13	2.56	0.16

**Table 3:** Permeation properties of fabricated membranes.

GOwt%	Jw (L/m^2^h)	Js (g/m^2^h)	Js/Jw g/L	A (Lm^−2^ h^−1^bar^−1^)	B (Lm^−2^h^−1^)	B/A (bar)	R%
CTA	17	3.3	0.19	10.96	1.93	0.17	85
CTA/0.3% GO	27	1.8	0.06	17.41	2.765	0.16	86
TFC	35	1.1	0.03	22.5	0.11	0.05	99.5

**Table 4:** Comparison between FO prepared membranes in the present work and previously reported FO membranes.

Polymer Weight %	GO Weight %	FluxL/m^2^h	DS	Citation
CA/GO	0.15	16	4 M glucose	[59]
CTA		15.2	2 M glucose	[22]
CTA/GO	0.6%	18.43	0.5 M NaCl	[22]
GO/Acetic Acid/CTA/CA	0.4%	33.5	1 M NH_4_Cl	[11]
CTA/Zn2Cl/Lactic acid	-	11.5	2 M glucose	[26]
CTA Prepared From Palm Fronds	-	17	2 M NaCl	Present work
CTA/GO Prepared From Palm Fronds	0.3% prepared from palm fronds	27	2 M NaCl	Present work
TFC/CTA	0.3% prepared from palm fronds	35	2 M NaCl	Present work

**Table 5:** Physico-chemical analysis of seawater.

Parameters	Unit	Results
pH	-	8.1
Sand content		N.D
Total solids	mg/L	42,702
Total dissolved solids	mg/L	42,643
Suspended solids	mg/L	59
Total hardness (CaCO_3_)	mg/L	7200
Calcium hardness (CaCO_3_)	mg/L	1040
Magnesium hardness (CaCO_3_)	mg/L	6160
Total alkalinity	mg/L	104
Bicarbonate	mg/L	80
Carbonate	mg/L	12
Hydroxide	mg/L	0
Methyl Orange alkalinity	mg/L	92
Phenolphthalein alkalinity	mg/L	12
Acidity	mg/L	12
Sulfate	mg/L	2200
Chloride (Cl^−^)	mg/L	25,000
Nitrite ($NO_{2}^{-}$-N)	mg/L	<0.005
Nitrate ($NO_{3}^{-}$-N)	mg/L	<0.05
Ammonium nitrogen	mgN/L	<0.01
Free carbon dioxide	mg/L	0.1
Phosphorus	mg/L	<0.01
Total silica	mg/L	<0.01
Non-reactive silica	mg/L	<0.01
Sodium (Na)	mg/L	19,200
Potassium (K)	mg/L	940
Calcium (Ca)	mg/L	416
Magnesium (Mg)	mg/L	1498

**Table 6:** Organic analysis of seawater.

Parameters	Unit	Results
Chemical Oxygen Demand	mgO_2_/L	14
Biological Oxygen Demand	mgO_2_/L	1.5
Oil & grease	mg/L	4.5
Total Organic Carbon	mgC/L	3.5

**Table 7:** Biological and microbiological examination of sea water.

Parameters	Unit	Results
Total bacterial count (22 °C)	Count/mL	2
Total bacterial count (37 °C)	Count/mL	1
Total Coliform	MPN-index/100 mL	N.D
Fecal Coliform	MPN-index/100 mL	N.D
Fecal Streptococci	MPN-index/100 mL	N.D
Blue Green Algae	Org/mL	N.D
Total Algal Count	Org/mL	40

**Table 8:** Characteristics of real saline water sample prior and post FO desalination of Neat CTA, CTA/0.3%GO, and TFC modified membranes and NaCl as the DS.

Ions	Concentrations (mg/L) of Sea Water before FO System.	R%of Effluent after FO System.		
		Neat CTA	CTA/0.3%GO	TFC Modified Membranes
Total Dissolved Solids	42,643	83	84.2	98.5
Calcium (Ca)	416	83	84	98
Magnesium (Mg)	1498	83	84.2	98.8
Sodium (Na)	19,200	90	93	99
Potassium (K)	940	84	84.3	99
Carbonate	12	83	84.2	99
Sulfate	2200	83	84.4	98
Chloride (Cl^−^)	25,000	83.3	85	99

**Table 9:** Physico-chemical analysis for seawater and permeate.

Parameters	Unit	Feed	Permeate	The Egyptian Ministerial Decree No. 458/2007
pH	-	8.1	7.5	6.5–8.5
Total Dissolved Solids	mg/L	42,643	640	1000
Suspended Solids	mg/L	59	3	-
Total Hardness (CaCO_3_)	mg/L	7200	110	500
Calcium Hardness (CaCO_3_)	mg/L	1040	21	350
Magnesium Hardness (CaCO_3_)	mg/L	6160	99	150
Total Alkalinity	mg/L	104	92	-
Bicarbonate	mg/L	92	92	-
Carbonate	mg/L	12	0	-
Hydroxide	mg/L	0	0	-
Sulfate	mg/L	2200	40	250
Chloride (Cl^−^)	mg/L	25,000	250	250
Nitrite ($NO_{2}^{-}$-N)	mg/L	<0.005	<0.005	0.2
Nitrate ($NO_{3}^{-}$-N)	mg/L	<0.05		45
Ammonium Nitrogen	mgN/L	<0.01	<0.01	-
Free Carbon Dioxide	mg/L	0.1	N.D	-
Phosphorus	mg/L	<0.01	<0.01	-
Total Silica	mg/L	<0.01	<0.01	-
Non reactive Silica	mg/L	<0.01	<0.01	-
Sodium (Na)	mg/L	19,200	186	200
Potassium (K)	mg/L	940	7	-
Calcium (Ca)	mg/L	416	9	-
Magnesium (Mg)	mg/L	1498	18	-

**Table 10:** Organic analysis for seawater and permeate.

Parameters	Unit	Feed	Permeate	The Egyptian Ministerial Decree No. 458/2007
Chemical Oxygen Demand	mgO_2_/L	14	2	-
Biological Oxygen Demand	mgO_2_/L	1.5	N.D	-
Oil & grease/’	mg/L	4.5	0.12	-
Total Organic Carbon	mgC/L	3.5	0.4	-

## Data Availability

No applicable.
